# Development of a Simple Direct and Hot-Start PCR Using *Escherichia coli*-Expressing *Taq* DNA Polymerase

**DOI:** 10.3390/ijms241411405

**Published:** 2023-07-13

**Authors:** Sun Ju Lee, Sang-Yong Park, Kwang-Ho Lee, Min-Woo Lee, Chae-Yeon Yu, Jaeyoung Maeng, Hyeong-Dong Kim, Suhng Wook Kim

**Affiliations:** 1 Department of Health and Safety Convergence Science, Graduate School, Korea University, 145 Anam-ro, Seoul 02841, Republic of Korea; 2 L-HOPE Program for Community-Based Total Learning Health Systems, Korea University, 145 Anam-ro, Seoul 02841, Republic of Korea; 3 Department of Laboratory Medicine, ASAN Medical Center, 88 Olympic-ro 43-gil, Songpa-gu, Seoul 05505, Republic of Korea; 4 Graduate School of Particulate Matter Specialization, Korea University, 145 Anam-ro, Seoul 02841, Republic of Korea

**Keywords:** *Taq* DNA polymerase, hot-start PCR, direct PCR, whole blood

## Abstract

*Taq* DNA polymerases have played an important role in molecular biology for several years and are frequently used for polymerase chain reaction (PCR); hence, there is an increasing interest in developing a convenient method for preparing *Taq* DNA polymerase for routine use in laboratories. We developed a method using *Escherichia coli* (*E. coli*) that expresses thermostable *Taq* DNA polymerase directly in the PCR without purification. The *Taq* gene was transformed into *E. coli* and expressed. After overnight incubation and washing, *E. coli*-expressing *Taq* DNA polymerase (EcoliTaq) was used as the DNA polymerase without purification. EcoliTaq showed activity comparable to that of commercial DNA polymerase and remained stable for 3 months. With a high-pH buffer containing 2% Tween 20 and 0.4 M trehalose, EcoliTaq facilitated direct PCR amplification from anticoagulated whole blood samples. EcoliTaq exhibited good performance in allele-specific PCR using both purified DNA and whole blood samples. Furthermore, it proved to be useful as a DNA polymerase in hot-start PCR by effectively minimizing non-specific amplification. We developed a simple and cost-effective direct and hot-start PCR method in which EcoliTaq was used directly as a PCR enzyme, thus eliminating the laborious and time-consuming steps of polymerase purification.

## 1. Introduction

The thermostable *Taq* DNA polymerase is commonly used in the polymerase chain reaction (PCR) for the detection of infectious agents in patient samples, genetic analysis, tumor diagnostics, research, and forensics [1,2,3]. However, *Taq* DNA polymerase is expensive, and hence, it is necessary to develop a simple method by which an individual laboratory can easily and routinely procure large quantities of *Taq* DNA polymerase.

Most thermostable DNA polymerases, including *Taq* DNA polymerase, are expressed as recombinant proteins in *Escherichia coli* (*E. coli*) harboring expression vectors. Polymerase purification is typically necessary during PCR [4]. However, PCR without polymerase purification is a rapid and simple approach involving the direct use of *E. coli* harboring a thermostable DNA polymerase as a PCR enzyme, which eliminates the tedious steps of protein purification. Therefore, the development of a simple and cost-effective PCR protocol using live *E. coli*-expressing *Taq* DNA polymerase (EcoliTaq) without polymerase purification is of practical significance and commercial interest.

PCR analysis of clinical samples, such as whole blood, to identify microbial infections or other genetic information usually involves sample purification and DNA extraction processes [5,6,7]. This is because unpurified whole blood contains inhibitors, such as anticoagulants [7,8] or hemoglobin [9], which can reduce PCR efficiency. Moreover, DNA extraction is a laborious and time-consuming process, which poses a significant disadvantage [10]. Therefore, there is a need for a direct PCR method involving an effective suppression of inhibitors and direct utilization of samples. In this study, we developed a direct PCR method that utilizes EcoliTaq and blood as DNA sources, eliminating the need for DNA purification.

Various efforts have been made to improve PCR technology in terms of the yield, specificity, and length of the amplified DNA. A major problem with PCR is that undesired DNA synthesis can sometimes occur during the PCR setup because of mispriming and the formation of a primer dimer. Hot-start PCR was developed to prevent unwanted DNA synthesis during PCR [11]. Hot-start PCR is based on the blocking of DNA synthesis at the ambient temperature, which has been achieved using chemical modification of the polymerase [12,13], antibodies [14,15,16], aptamers [17,18], wax [19], reaction buffer [20], special primers [21,22], and cold-sensitive polymerase mutants [23].

In this study, we developed a simple hot-start PCR protocol using EcoliTaq as the polymerase. This novel method reduces the activity of *Taq* DNA polymerase by physically separating *Taq* DNA polymerase, which exists within the *E. coli* membrane, from primers and other PCR reagents at lower temperatures. After the initial denaturation step, the cell wall and membrane of *E. coli* were destroyed, and *Taq* DNA polymerase freely participated in the amplification process.

## 2. Results

### 2.1. Activity of EcoliTaq in Commercial PCR Buffer

**Figure 1:**
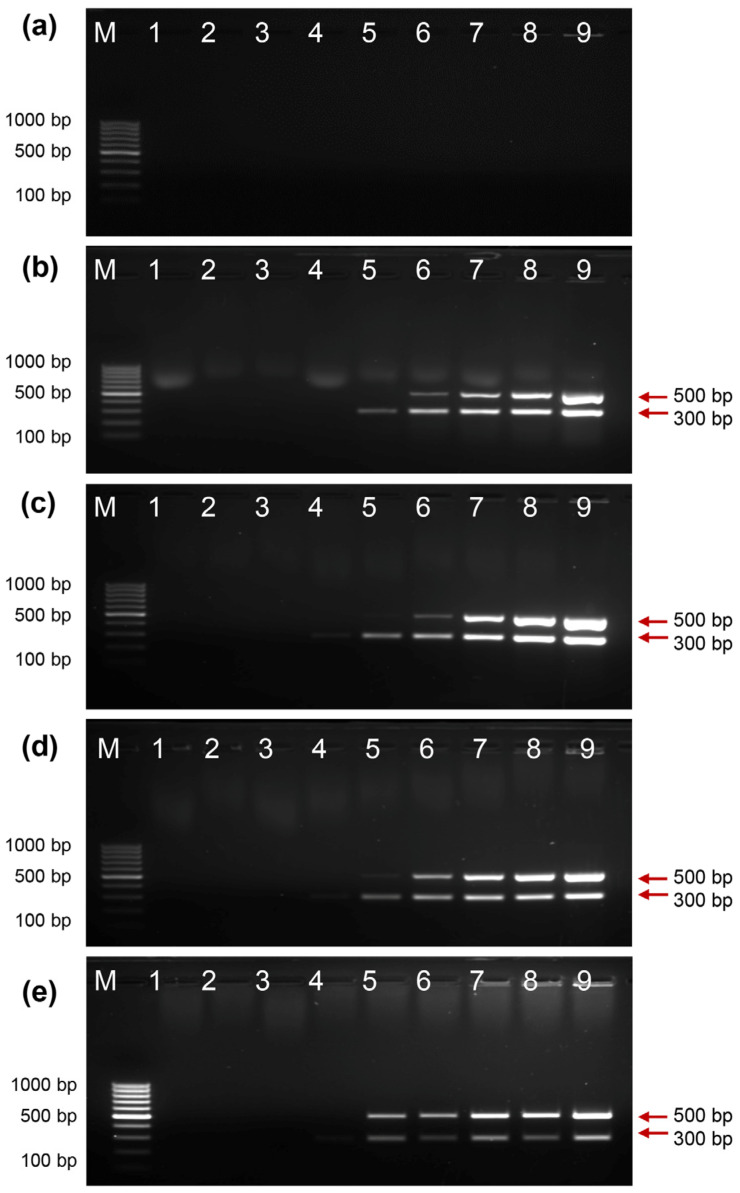
Analysis of EcoliTaq activity in commercial PCR buffer with or without Tween 20. The expected amplified DNA fragments are marked with arrows. A 0% (**a**), 1% (**b**), 2% (**c**), 5% (**d**), and 10% (**e**) concentration of Tween 20 was incorporated into a commercial PCR buffer. The EcoliTaq was diluted in the following ratios: 1:256 (lane 1), 1:128 (lane 2), 1:64 (lane 3), 1:32 (lane 4), 1:16 (lane 5), 1:8 (lane 6), 1:4 (lane 7), 1:2 (lane 8), and 1:1 (lane 9, OD_600_ = 0.8). M, 100 bp DNA ladder (100 bp–1000 bp).

Multiplex PCR amplification was used to determine the activity of EcoliTaq. After culturing overnight and washing, the EcoliTaq was adjusted to a final optical density of 0.8 at 600 nm (OD_600_). PCR amplification using EcoliTaq as the only *Taq* DNA polymerase source was suppressed when a commercial PCR buffer was used (Figure 1a). However, as shown in Figure 1b–e, clear gel bands were observed when Tween 20 was added. The addition of 2% Tween 20 to the commercial PCR buffer resulted in the best amplification of the two lambda genomic targets (500 and 300 bp).

### 2.2. Activity of EcoliTaq Compared with That of Commercial Taq DNA Polymerase

**Figure 2:**
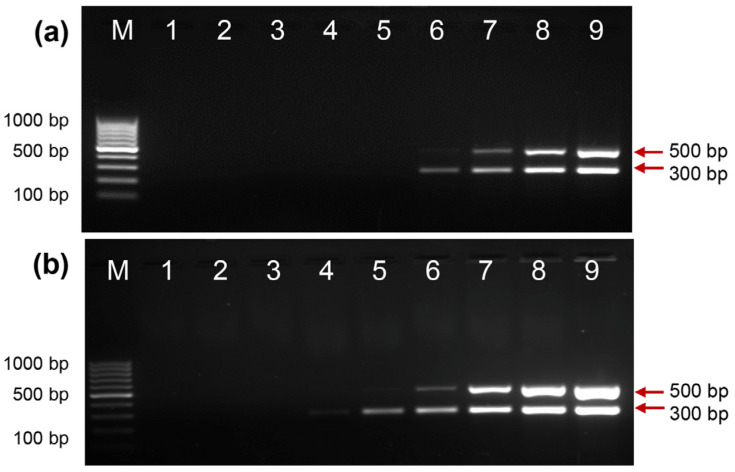
Analysis of the activity of EcoliTaq compared with that of commercial Taq DNA polymerase. (**a**) Commercial Taq DNA Polymerase was diluted in the following ratios: 1:256 (lane 1), 1: 128 (lane 2), 1:64 (lane 3), 1:32 (lane 4), 1:16 (lane 5), 1:8 (lane 6), 1:4 (lane 7), 1:2 (lane 8), and 1:1 (lane 9, 0.5 unit), (**b**) The EcoliTaq was diluted in the following ratios: 1:256 (lane 1), 1:128 (lane 2), 1:64 (lane 3), 1:32 (lane 4), 1:16 (lane 5), 1:8 (lane 6), 1:4 (lane 7), 1:2 (lane 8), and 1:1 (lane 9, OD_600_ = 0.8). M, 100 bp DNA ladder (100 bp–1000 bp).

We determined the approximate units of EcoliTaq using a multiplex PCR amplification assay by titration against commercial *Taq* DNA Polymerase. The amplified products obtained using 1:2-diluted EcoliTaq (Figure 2b, lane 8) were nearly equivalent to the amplified products using 0.5 units of commercial *Taq* DNA Polymerase (Figure 2a, lane 9).

### 2.3. Activity of EcoliTaq at Different Storage Temperatures

**Figure 3:**
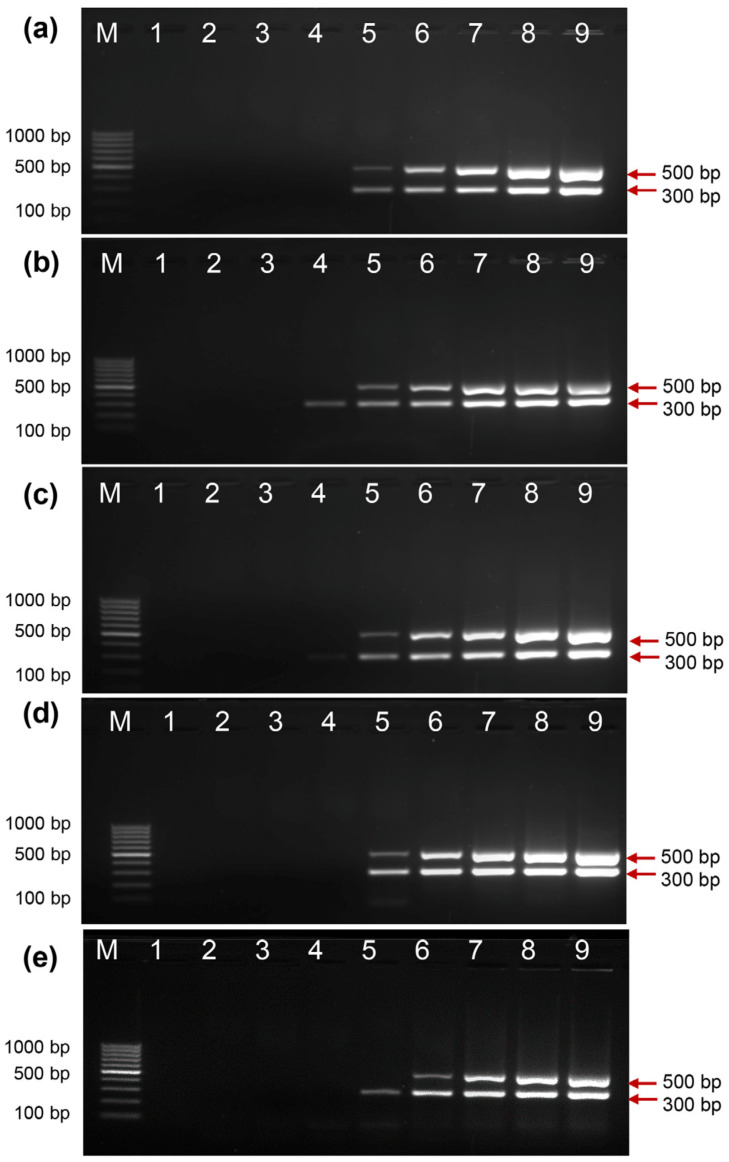
Analysis of EcoliTaq activity at different storage temperatures. EcoliTaq stored (**a**) −80 °C, (**b**) −20 °C, (**c**) 4 °C, (**d**) 25 °C, and (**e**) 37 °C for 3 months was used. The EcoliTaq was diluted in the following ratios: 1:256 (lane 1), 1:128 (lane 2), 1:64 (lane 3), 1:32 (lane 4), 1:16 (lane 5), 1:8 (lane 6), 1:4 (lane 7), 1:2 (lane 8), and 1:1 (lane 9, OD_600_ = 0.8). M, 100 bp DNA ladder (100 bp–1000 bp).

We measured the duration for which the activity of EcoliTaq was maintained at different storage temperatures. The PCR amplification assay using EcoliTaq stored at −80 °C (Figure 3a), −20 °C (Figure 3b), 4 °C (Figure 3c), 25 °C (Figure 3d), and 37 °C (Figure 3e) for 3 months showed negligible differences in PCR yields.

### 2.4. Activity of EcoliTaq in the Presence of Whole Blood

**Figure 4:**
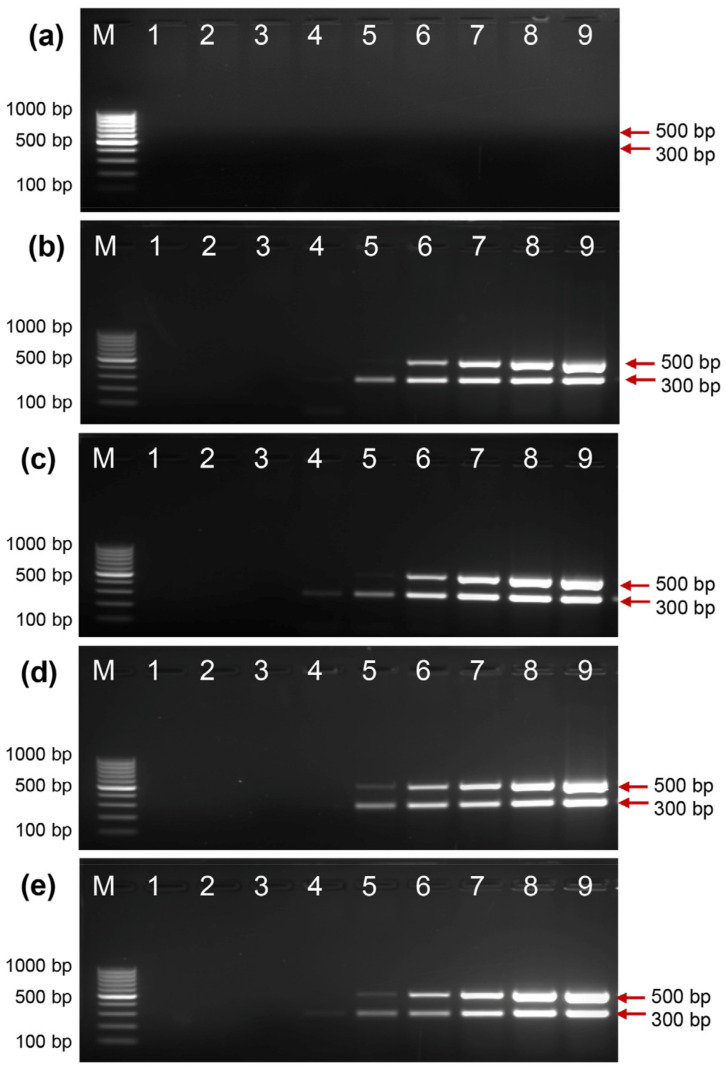
Analysis of EcoliTaq activity in the presence of whole blood. Different concentrations of trehalose ((**a**) 0 M, (**b**) 0.1 M, (**c**) 0.2 M, (**d**) 0.4 M, and (**e**) 0.6 M) was added to PCR reaction mixtures. The EcoliTaq was diluted in the following ratios: 1:256 (lane 1), 1:128 (lane 2), 1:64 (lane 3), 1:32 (lane 4), 1:16 (lane 5), 1:8 (lane 6), 1:4 (lane 7), 1:2 (lane 8), and 1:1 (lane 9, OD_600_ = 0.8). M, 100 bp DNA ladder (100 bp–1000 bp).

The activity of EcoliTaq in the presence of whole blood was determined using a multiplex PCR amplification assay. Using a commercial PCR buffer, no PCR amplification was obtained with 1 μL of whole blood (Figure 4a), which indicated the presence of PCR inhibitors in the whole blood. Trehalose has been previously identified as a highly effective PCR enhancer that protects *Taq* DNA polymerase from the negative effects of blood inhibitors [24]. When trehalose was added at different concentrations, two clear bands in the PCR gel were observed (Figure 4b–e). The highest activity was observed with 0.4 M trehalose; hence, 0.4 M trehalose was used for subsequent assays.

### 2.5. Activity of EcoliTaq in the Presence of Whole Blood Containing Three Anticoagulants at Different pH Values

**Figure 5:**
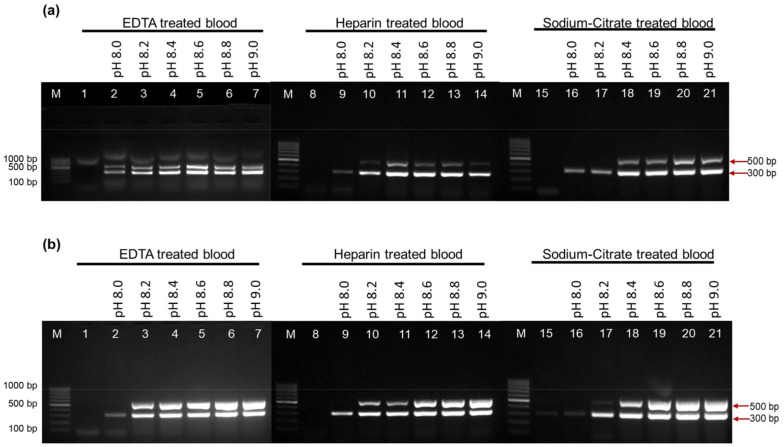
Analysis of EcoliTaq activity using whole blood treated with three anticoagulants at different pH. PCR was performed with 1 μL of anticoagulant-treated whole blood containing EDTA (lanes 1–7), heparin (lanes 8–14), and sodium citrate (lanes 15–21). (**a**) A bicine-based PCR buffer was used with pH values of 8.0 (lane 2), 8.2 (lane 3), 8.4 (lane 4), 8.6 (lane 5), 8.8 (lane 6), and 9.0 (lane 7), and a commercial PCR buffer was used in lane 1. (**b**) A tricine-based PCR buffer was used with pH values of 8.0 (lane 2), 8.2 (lane 3), 8.4 (lane 4), 8.6 (lane 5), 8.8 (lane 6), and 9.0 (lane 7), and a commercial PCR buffer was used in lane 1. M, 100 bp DNA marker (100 bp–1000 bp).

We developed a reliable PCR buffer to enhance the performance of EcoliTaq. The activity of EcoliTaq was determined by a multiplex PCR amplification assay using whole blood containing three anticoagulants: EDTA, heparin, and sodium citrate, which are commonly used in clinical blood samples. We tested various concentrations of bicine- or tricine-based buffers at pH 8.0–9.0. Compared with the commercial PCR buffer (Figure 5a,b, lane 1,8,15), the PCR buffer containing 2% Tween 20 and 0.4 M trehalose at a high pH exhibited a superior performance in amplifying the amplicons and suppressing the PCR inhibitors in the whole blood sample. As shown in Figure 5b, the optimal pH range of the tricine buffer for successful PCR was 8.4–9.0. We chose the tricine-based buffer (pH 8.6) containing 2% Tween 20 and 0.4 M trehalose (Figure 5b, lane 19) for PCR analysis.

### 2.6. Direct PCR Detection of Salmonella and Shigella in Whole Blood

**Figure 6:**
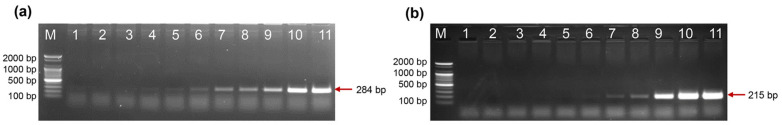
PCR amplification for the detection of infectious microorganisms in whole blood using EcoliTaq. (**a**) The PCR amplification of the invA gene for S. typhimurium was performed, and the following concentrations were used: lane 1, 12 CFU/mL; lane 2, 25 CFU/mL; lane 3, 50 CFU/mL; lane 4, 100 CFU/mL; lane 5, 200 CFU/mL; lane 6, 400 CFU/mL; lane 7, 800 CFU/mL; lane 8, 1600 CFU/mL; lane 9, 3200 CFU/mL; lane 10, 6400 CFU/mL; and lane 11, 12,800 CFU/mL. (**b**) The PCR amplification of the virA gene for S. flexneri was performed, and the following concentrations were used: lane 1, 20 CFU/mL; lane 2, 40 CFU/mL; lane 3, 80 CFU/mL; lane 4, 160 CFU/mL; lane 5, 320 CFU/mL; lane 6, 640 CFU/mL; lane 7, 1280 CFU/mL; lane 8, 2560 CFU/mL; lane 9, 5120 CFU/mL; lane 10, 10,240 CFU/mL; and lane 11, 20,480 CFU/mL. M, 100 bp DNA marker (100 bp–2000 bp).

We directly diagnosed the infectious microorganisms in the whole blood samples without DNA purification and used 1 μL of whole blood inoculated with live *Salmonella typhimurium* and *Shigella flexneri* as PCR templates. We conducted direct PCR using EcoliTaq and a tricine-based buffer with a pH of 8.6, which contained 2% Tween 20 and 0.4 M trehalose. This resulted in the successful amplification of a 284 bp fragment of the *invA* gene for *S. typhimurium* and a 215 bp fragment of the *virA* gene for *S. flexneri* (Figure 6). The minimum number of cells required for the detection of *S. typhimurium* by direct PCR using EcoliTaq was 200 CFU/mL (Figure 6a, lane 5). The minimum number of cells required for the detection of *S. flexneri* by direct PCR using EcoliTaq was 640 CFU/mL (Figure 6b, lane 6).

### 2.7. HLA-B27 Genotyping Using Clinical Samples

**Figure 7:**
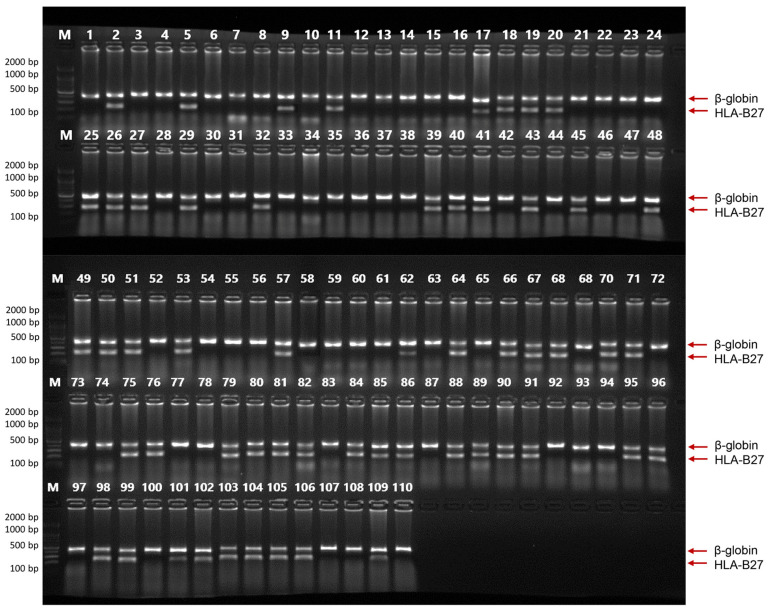
HLA-B27 genotyping using EcoliTaq. The PCR product specific for the internal control, β-globin, was observed at 268 bp. The PCR product specific for HLA-B27 positive samples was observed at 136 bp. M, 100 bp DNA marker (100 bp–2000 bp).

We compared the HLA-B27 genotyping results obtained using the EcoliTaq with that obtained using a commercial DNA polymerase incorporated into an HLA-B27 genotyping kit for clinical samples. We used 55 positive and 55 negative DNA samples from patients who underwent HLA-B27 genotyping at Seoul Asan Medical Center between June and September 2022, with the approval of the Institutional Review Board of Asan Medical Center (IRB No. S2022-2085-0002). When HLA-B27 genotyping was performed on 55 positive and 55 negative samples, the results obtained using EcoliTaq were found to be 100% concordant with those obtained using the commercial DNA polymerase included in the HLA-B27 genotyping kit (Figure 7).

### 2.8. Direct Allele-Specific PCR Using DNA and Whole Blood Samples

**Figure 8:**
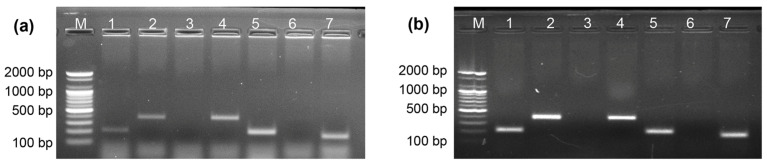
Performance of EcoliTaq in ASPCR for ABO genotyping was analyzed using purified DNA (**a**) and whole blood (**b**). Lanes 1–7 correspond to the PCR reaction numbers in Table 1. M, 100 bp DNA ladder (100 bp–2000 bp).

Allele-specific PCR (ASPCR) is a powerful tool for discriminating between alleles arising from single-base substitutions or deletions. Allele discrimination in ASPCR relies on the inability of *Taq* DNA polymerase, which lacks proofreading activity, to extend a primer when the 3′ nucleotide is not complementary to the DNA template. In this study, we used ASPCR with EcoliTaq to perform ABO genotyping of both DNA and whole blood samples. In Figure 8, as expected, for genotype BO, no amplification was observed in lane 3 (164 bp) or 6 (381 bp), whereas successful amplification of the target amplicons was achieved in lanes 1 (205 bp), 2 (381 bp), 4 (381 bp), 5 (205 bp), and 7 (164 bp) (Table 1). These results agree with previous reports on ASPCR [25], indicating the feasibility of using EcoliTaq as a DNA polymerase in ASPCR.

### 2.9. Activity of EcoliTaq in Hot-Start PCR

**Figure 9:**
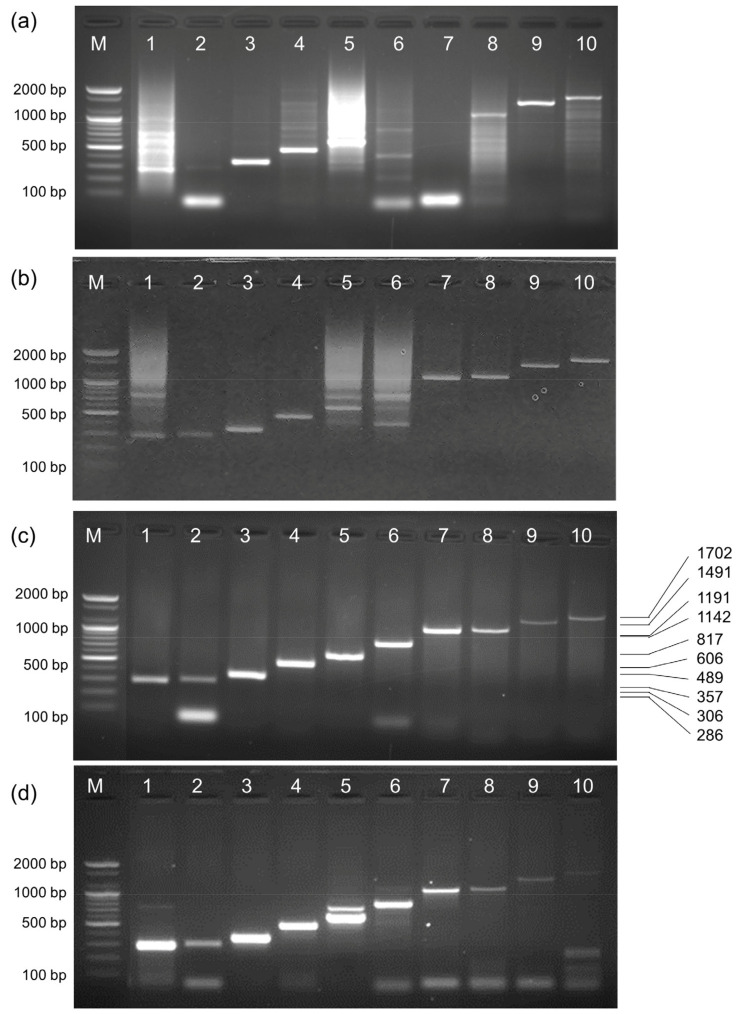
Hot-start PCR amplification to detect 10 targets of the P53 gene. The amplification was performed using (**a**) a commercial *Taq* DNA polymerase with DNA as the template, (**b**) a commercial HotStart *Taq* DNA polymerase with DNA as the template (**c**) EcoliTaq with DNA as the template, and (**d**) EcoliTaq with whole blood as the template. Lane 1–10: the used primers and their corresponding expected amplicon size are presented in Table 2. M, 100 bp DNA ladder (100 bp–2000 bp).

We developed a simple hot-start method using EcoliTaq. This novel method uses *Taq* DNA polymerase surrounded by *E. coli* membrane to sequester primers and other PCR reagents at low temperatures, rendering them unavailable for extension by the polymerase. *Taq* DNA polymerase was easily released by heating at 95 °C for 5 min to lyse *E. coli* and denature the released proteins, except for polymerase, followed by PCR. An experiment was conducted to evaluate the performance of EcoliTaq as a DNA polymerase in a hot-start PCR using 10 primer pairs targeting the single-copy p53 gene. When a commercial *Taq* DNA polymerase without a hot-start feature was used with DNA as the template, multiple non-specific bands were observed (Figure 9a). It can be seen that when a commercial *Taq* DNA polymerase with hot-start function was used with the DNA template, non-specific bands appeared, except lane 1, 5, 6 (Figure 9b). However, when EcoliTaq was used with DNA as the template, a clear and distinct pattern of 10 different-sized target bands from the single-copy p53 gene was observed (Figure 9c). Notably, even when whole blood was used as a template in the hot-start PCR with EcoliTaq, non-specific PCR products were effectively eliminated (Figure 9d). These results indicated that the EcoliTaq effectively blocks non-specific product formation before thermal cycling and acts as a DNA polymerase in a hot-start PCR.

## 3. Discussion

A simple and cost-effective PCR method was developed using *E. coli* that expresses a thermostable *Taq* DNA polymerase. After overnight culture and washing, EcoliTaq was fully functional as a DNA polymerase, showing that EcoliTaq can be directly employed in PCR when an appropriate buffer is used. As shown in Figure 3, EcoliTaq could maintain the *Taq* DNA polymerase stability at ambient temperature, making it an attractive option for *Taq* DNA polymerase storage. Therefore, this method can be effectively utilized in laboratories or diagnostic facilities under less-than-ideal environmental conditions. The most remarkable feature of the PCR method involving EcoliTaq is that it can be conducted anywhere, provided that *E. coli* can be cultured using readily available means.

We prepared a buffer with a high pH as it is used for the direct PCR analysis of whole blood to weaken the interaction between the inhibitors and genomic DNA [26]. Our buffer contained Tween 20, which can improve DNA sequencing by reducing the frequency of nonspecific bands [27]; moreover, Tween 20 could facilitate the lysis of *E. coli* cells [28]. Our buffer also contained trehalose, which promotes the activity of thermostable enzymes at high temperatures [29]. Trehalose can also enhance the yield of the amplified product, especially in PCR with blood inhibitors [24], possibly by lowering the template melting temperature and eliminating secondary structures [30,31].

Commonly used hot-start PCR techniques target the polymerase by silencing its activity before the initial denaturation step using a blocking antibody or chemical modification [32]. Neutralizing antibodies that can block the activity of *Taq* polymerase at low temperatures and activate it at high temperatures are commercially available [15]. However, blocking antibodies are expensive, sensitive to temperature, and must be shipped and stored at low temperatures. A chemically modified *Taq* polymerase that is inactive at low temperatures and is activated upon heating at 95 °C for 10–20 min has also been used in hot-start PCR [12]. However, the removal of the chemical blocking group on the polymerase typically requires an initial denaturation time > 10 min, which causes heat damage to the DNA samples. The ideal solution is a cost-effective hot-start method that allows easy handling and a shorter initial denaturation time. EcoliTaq offers several advantages, including ease of handling and storage. Additionally, it does not require pre-reactivation before it initiates amplification and is available at a low cost. EcoliTaq was produced using *E. coli*, and the heat-labile nature of the *E. coli* membrane allowed for an initial denaturation time of 5 min. This reduced the likelihood of heat-induced DNA damage.

Another advantage is that because our method directly used *E. coli*, a variety of thermostable polymerases can be used in hot-start PCR if they are expressed in *E. coli*. This method is extremely convenient and inexpensive because polymerase purification, which is a laborious and tedious procedure, is not required. In addition, the speed and efficiency of this method, along with the availability of commercial recombinant plasmids for various thermostable enzymes, suggests that this method can be adapted to various thermostable enzyme-requiring reactions from different sources.

Using EcoliTaq, we developed a direct PCR technique that enables the direct amplification of DNA from whole blood without prior DNA extraction. This reduces time and cost and eliminates the need for complex processes and toxic chemicals used in DNA purification. Direct PCR methods commonly employ genetically modified DNA polymerases with a higher tolerance to inhibitors and proprietary additives, such as PCR enhancers in the reaction buffer [31,33,34,35]. Although these methods commonly use purified *Taq* DNA polymerase, our direct PCR method used unpurified *E. coli*, making it a low-cost alternative.

One major drawback of using EcoliTaq is the potential for contamination of the PCR reactions by the genome of *E. coli* due to the use of live *E. coli*. However, it should be noted that most commercial *Taq* DNA polymerase preparations already contain microbial DNA contaminants, which are present in the DNA polymerase preparations. The removal of contaminants from the DNA of the enzyme source during 16S rRNA analysis and purification of *Taq* formulations can be problematic [36]. Therefore, contamination can be addressed by recognizing the contaminated situation and conducting experiments accordingly. One approach is to conduct a sensitivity test using sequencing or restriction enzyme digestion of the target band to evaluate any potential interference in primer design associated with this method.

The *Taq* DNA polymerase is homologous to the *E. coli* DNA polymerase I [37], so contamination with the *E. coli* DNA polymerase I is a possible risk. However, it should be noted that the *E. coli* DNA polymerase I exhibits an optimal activity at 37 °C, but becomes inactive after 5 min of exposure to 95 °C. Therefore, the *E. coli* DNA polymerase I does not pose a substantial concern in PCR with EcoliTaq.

## 4. Materials and Methods

### 4.1. Materials

Human whole blood treated with three anticoagulants (EDTA, heparin, and sodium citrate) was purchased from KOMA BIOTECH (Seoul, Republic of Korea). Human genomic DNA was isolated from the blood using a conventional DNeasy Blood and Tissue Kit (Qiagen, Germantown, MD, USA). *Salmonella typhimurium* (KCCM 40253) and *Shigella flexneri* (KCCM 40948) were obtained from the Korean Culture Center for Microorganisms. The HLA-B27 PCR kit was procured from BioSewoom (Seoul, Republic of Korea).

### 4.2. Preparation of EcoliTaq

The *Taq* DNA polymerase gene was cloned into the expression vector pKK223-3, and the resulting recombinant plasmid was transformed into *E. coli*. The transformed *E. coli* was inoculated into 10 mL of Lauria Bertaini broth media and incubated at 37 °C in a shaking incubator for 3–5 h, until the absorbance of the culture at OD_600_ was 0.4–0.6. The OD_600_ value was measured at a wavelength of 600 nm using a disposable plastic cuvette with a path length of 1 cm (Ratiolab, Dreieich, Germany) on a spectrophotometer (Evolution 60S, Thermo Fisher Scientific, MA, USA). When the absorbance of the culture at OD_600_ was 0.4–0.6, 0.1 mM isopropyl-β-D-thiogalactopyranoside (Thermo Fisher Scientific, MA, USA) solution was added. The mixture was incubated for 18 h at 37 ℃ in a shaking incubator. After incubation, the mixture was centrifuged at 1072× *g* (4000 rpm) for 10 min. The supernatant was discarded, and the pellet was washed twice with 5 mL of distilled water (DW). The absorbance of the washed EcoliTaq mixture was adjusted to 0.8 at 600 nm through dilution with DW. Each mixture was kept at −80 °C, −20 °C, 4 °C, 20 °C, and 37 °C. Glycerol (10%) was added to samples stored at −80 °C.

### 4.3. PCR Amplification for Assay of EcoliTaq Activity

EcoliTaq activity was determined using a PCR amplification assay through titration against a commercial DNA polymerase. For the enzyme activity assay, a commercial DNA polymerase was diluted appropriately with storage buffer before adding it to the reaction mixtures, and EcoliTaq was diluted with DW. The reaction mixtures consisted of a 1× PCR buffer with or without Tween 20 and trehalose, 0.25 mM dNTP, 0.25 μM of the 2 primer pairs (5′-GATGAGTTCGTGTCCGTACAACTGG-3′ and 5′-GGTTATCGAAATCAGCCACAGCGCC-3′; 5′-TTCAGGCGGCGCATTTTTATT-3′ and 5′-ACGTCGATGACATTTGCCGTA-3′) that amplify 500-bp and 300-bp fragments corresponding to nucleotides 7131–7630 and 30,537–30,836 of the bacteriophage lambda genome [33], respectively, and 1 ng of the lambda genomic DNA or 1 μL blood. Amplification involved the initial denaturation at 95 °C for 5 min, followed by 30 cycles of denaturation at 94 °C for 30 s, primer annealing at 60 °C for 30 s, extension at 72 °C for 30 s, and the final extension at 72 °C for 10 min. PCR was performed using the Rotor-Gene 6000 system (Corbett Research, Sydney, Australia). After the PCR was completed, 10 μL aliquots of the PCR products were stained with ethidium bromide and analyzed by electrophoresis on a 1.5% agarose gel.

### 4.4. Detection of Salmonella spp. and Shigella spp. in Whole Blood Samples

PCR amplification was performed in a 20 μL mixture of a 1× tricine-based buffer (pH 8.6) containing 2% Tween 20 and 0.4 M trehalose, 0.25 mM dNTP, and 1 μL of whole blood and diluted bacterial mixture. Primers (0.25 Μm) specific for the *invA* gene (284 bp) were used for *S. typhimurium* [38], and primers (0.25 μM) specific for the *virA* gene (215 bp) were used for *S. flexneri* [39]. Amplification involved initial denaturation at 95 °C for 5 min followed by 30 cycles of denaturation at 94 °C for 30 s, primer annealing at 60 °C for 30 s, extension at 72 °C for 30 s, and the final extension at 72 °C for 10 min. PCR was performed using the same Rotor-Gene 6000 system and the results were analyzed in the same manner as that of the PCR amplification assay.

### 4.5. HLA-B27 Genotyping Using Clinical Samples

PCR was conducted using a mixture comprising of 7 μL of the master mixture consisting of PCR mixture from the HLA-B27 PCR kit and EcoliTaq, and 3 μL of DNA sample, ranging from 26 to 189 ng/μL. Amplification involved an initial denaturation at 95 °C for 5 min, followed by 30 cycles of denaturation at 94 °C for 30 s, primer annealing at 65 °C for 30 s, extension at 72 °C for 30 s, and the final extension at 72 °C for 5 min. A 2% agarose gel was prepared by adding 3.5 μL of DNA staining dye (RedSafe, INtRON, Seongnam, Republic of Korea) to 70 mL of gel solution. The PCR products (5 μL) were loaded onto a gel and subjected to electrophoresis at 150 V for approximately 15 min to visualize the bands.

### 4.6. ASPCR

ASPCR was performed with a 20 μL reaction mixture of a 1× tricine-based buffer (pH 8.6) containing 2% Tween 20 and 0.4 M trehalose for EcoliTaq, 0.25 mM dNTP, and 3 ng of human DNA or 1 μL of EDTA-treated whole blood. Then, 5 μL of EcoliTaq OD_600_ = 0.8 was used as the DNA polymerase. The ABO genotype was determined using 0.25 μM of each primer pair listed in Table 1 (reproduced from reference [25]). The cycling conditions for the ASPCR were an initial denaturation for 5 min at 95 °C, followed by 35 cycles of 40 s at 95 °C, 40 s at 60 °C, 40 s at 72 °C, and an additional 5 min at 72 °C for the final elongation. After the reaction, 10 μL of the PCR mixture was electrophoresed on a 1.5% agarose gel stained with ethidium bromide.

### 4.7. Hot-Start PCR

A hot-start PCR was done in a 20 μL mixture containing a 1× tricine-based buffer (pH 8.6) containing 2% Tween 20 and 0.4 M trehalose for EcoliTaq, a 1× commercial PCR buffer for commercial DNA polymerase, 0.25 mM dNTP, and 3 ng of human DNA or 1 μL EDTA-treated whole blood. Then, 5 μL of EcoliTaq (OD_600_ = 0.8) and 0.5 U of commercial DNA polymerase with or without hot-start features was used as DNA polymerase, and 0.25 μM of each of the 2 primer pairs (Table 2) was used to amplify 1 of the p53 gene sequences [25]. Amplification involved initial denaturation at 95 °C for 5 min, followed by 30 cycles of denaturation at 94 °C for 30 s, primer annealing at 60 °C for 30 s, extension at 72 °C for 60 s, and the final extension at 72 °C for 10 min. PCR was performed using the same Rotor-Gene 6000 system and the results were analyzed in the same manner as those of the PCR amplification assay.

## 5. Conclusions

We developed a simple, direct, and hot-start PCR method for routine use. This alternative PCR method offered significant advantages in terms of convenience and cost-effectiveness. It used an *E. coli*-expressing thermostable *Taq* DNA polymerase directly as the PCR enzyme, thus eliminating the laborious and time-consuming steps involved in polymerase purification. To the best of our knowledge, this is the first report demonstrating the efficacy of *E. coli* as an enzyme source in practical PCR procedures.

## Figures and Tables

**Table 1:** Primers for ABO genotyping.

PCR Reaction	Fragment Size (bp)	Primer Pair	Allele Specificity
1	205	261G: 5′-GCAGTAGGAAGGATGTCCTCGTGtTG-3′	*A101*, *A102*, *B101*
		int6: 5′-AGACCTCAATGTCCACAGTCACTCG-3′	
2	381	467C: 5′-CCACTACTATGTCTTCACCGACCAtCC-3′	*A101*, *O01*, *O02*
		803G: 5′-CACCGACCCCCCGAAGAtCC-3′	
3	164	297A: 5′-CCATTGTCTGGGAGGGCcCA-3′	*A101*, *A102*, *O01*
		int6: 5′-AGACCTCAATGTCCACAGTCACTCG-3′	
4	381	467C: 5′-CCACTACTATGTCTTCACCGACCAtCC-3′	*B101*
		803C: 5′-CACCGACCCCCCGAAGAtCG-3′	
5	205	261A: 5′-GCAGTAGGAAGGATGTCCTCGTGtTA-3′	*O01*, *O02*
		int6: 5′-AGACCTCAATGTCCACAGTCACTCG-3′	
6	381	467T: 5′-CCACTACTATGTCTTCACCGACCAtCT-3′	*A102*
		803G: 5′-CACCGACCCCCCGAAGAtCC-3′	
7	164	297G: 5′-CCATTGTCTGGGAGGGCcCG-3′	*B101*, *O02*
		int6: 5′-AGACCTCAATGTCCACAGTCACTCG-3′	

**Table 2:** Hot-start PCR primers for p53 gene amplification.

PCR Reaction	Fragment Size (bp)	Primer Sequence
1	286	5′-GGCGACAGAGCGAGATTCCA-3′
		5′-GGGTCAGCGGCAAGCAGAGG-3′
2	306	5′-GAGGTTCCTACAGGCACCTGCCCAG-3′
		5′-CAAAATCACCCCTCACAGTACTCTG-3′
3	357	5′-GACAAGGGTGGTTGGGAGTAGATG-3′
		5′-AGAGGAGCTGGTGTTGTTGG-3′
4	489	5′-TGTTCACTTGTGCCCTGACT-3′
		5′-GGAGGGCCACTGACAACCA-3′
5	606	5′-GGCGACAGAGCGAGATTCCA-3′
		5′-CACAAACACGCACCTCAAAG-3′
6	817	5′-GGCGACAGAGCGAGATTCCA-3′ 5′-AGAGGAGCTGGTGTTGTTGG-3′
7	1142	5′-GCTTTATCTGTTCACTTGTGCCC-3′ 5′-TGTGCAGGGTGGCAAGTGGC-3′
8	1191	5′-TGTTCACTTGTGCCCTGACT-3′ 5′-GGGTCAGCGGCAAGCAGAGG-3′
9	1491	5′-TGTTCACTTGTGCCCTGACT-3′ 5′-CACAAACACGCACCTCAAAG-3′
10	1702	5′-TGTTCACTTGTGCCCTGACT-3′
		5′-AGAGGAGCTGGTGTTGTTGG-3′

## Data Availability

Data contained within the article.
